# A Case of General Paresis

**Published:** 1873-12

**Authors:** J. B. Stonehouse

**Affiliations:** Assistant Physician Sanford Hall, Flushing, New York


*A Case of General Paresis.* By J. B. STONEHOUSE, JR., M.D., Assistant Physician Sanford Hall, Flushing, New York.

Certain diseases—which indeed properly belong to the domain of the specialist, but are in the earlier stages, when a proper appreciation of the symptoms is of the greatest importance, in the hands of the general practitioner—are seldom spoken of in the text-books or by the college instructors, while the medical journals only at wide intervals refer to them. One of these, general paresis, is peculiarly liable to a mistaken diagnosis, with the consequent damage to the patient's chances of recovery: especially is this the case when the paresis precedes the development of the *psychical* symptoms. A recent writer (Guy's Hospital Reports, 1870) argues that cases occur outside of the hospitals for the insane which deserve the title of general paresis as fully as those within. In accordance with this view, and with the hope of presenting to the readers of the *Practitioner* in a small space a general idea of the disease, with the views thereon of the recognized authorities, this article has been prepared. The case reported is one which was at no time in an asylum, and is therefore chosen for the text.

M. H., merchant, aged forty-three years, single, of good habits and of previous excellent health. The first symptoms observed by him, and for which he sought the advice of a physician, were a feeling of fullness of the head, with vertigo, an inability to perform any severe mental labor, and loss of memory. Two months later mental symptoms made their appearance. Patient declared he was not fitted for his business, and would ruin his partners. He said it had been decreed by Providence that he should fail if he continued in the business. After much deliberation he concluded to change his occupation, and selected the popular lecture-field for his future operations, expressing great faith in his oratorical powers, and displaying all that wonderful train of mental symptoms usual in the "*delire des grandeurs.*" Even while making these assertions a peculiar trembling of the lips, "as if about to burst into tears" (Maudsley,) often embarrassing his articulation very perceptibly, was noticed. This condition increased rapidly until he was unable to pronounce the *labials.* The right pupil was contracted;* paresis of the muscles of deglutition and expression soon took place; the use of the fingers, and later of the lower

* Hammond (Diseases of the Nervous System, page 369) quotes Austin (Pract. Acct. of General Paralysis, etc., London, 1859) as asserting that contraction of the right pupil is associated with melancholic delusions, and contraction of the left pupil with the more self-complacent fancies. This, however, cannot be supported.

extremities, became impaired. A failure of muscular power, with considerable spasmodic action after continued effort to grasp the instrument, was shown by the dynamometer. Muscles of the extremities responded to the electric stimulus. He was unable to dress himself without great effort. His gait at this time was very similar to that observed in cases of sclerosis of posterior columns of the spinal cord. A spasmodic action of the pulse (which varied from 90 to 100 per minute) was exhibited by the sphygmograph. Ophthalmic examination was made only during the first stages of the disease, without revealing any lesions of the optic nerve. No convulsions, comatose attacks, or maniacal exacerbations occurred during the course of the disease. From the end of the third month his mental powers steadily diminished into a condition of dementia. Urine and fæces passed involuntarily, and the patient would remain in contact with them until the sense of smell informed him of his condition. At this time there was anæsthesia of the greater portion of the cutaneous surface and of the mucous membranes, as far as could be noticed. This condition was not complete in the axillary and inguinal regions. Extreme loss of memory was observable. He seldom remembered an extravagant idea longer than the time occupied in uttering it. When asked his own name, in the later periods, he could not answer. During a brief absence of the nurse from the room death occurred from impaction of food in the fauces, owing to anæsthesia of these parts. From the appearance of the first symptoms to the day of death was five months and fifteen days.

Among the points of interest noticed in this case are the following:

1. The short duration of so typical a case. Hammond gives the average duration as three years.

2. The absence of any definite cause. The patient, as we have before stated, was a man of excellent habits. He was an active and consistent temperance man. An inquiry as to his past history revealed the fact that several years before the attack, during which time he enjoyed very good health, the patient had been severely mercurialized during a course of treatment for some liver trouble. Could this have produced a condition of the nervous system inviting the development of the disease?

*Synonyms.* General paresis, general paralysis of the insane, paresifying mental disease, paresis generalis, insania paresans, dementia paralytica, anoia paralytica, geisteskrankheit mit paralyse, paralysie generale, alienation ambitieuse avec paralysie incomplete, folie paralytique, delire des grandeurs.

As to the causation of general paralysis, no great certainty of opinion has been reached. Abuse of alcohol, the narcotics, etc., venereal excesses, and many other habits and conditions, have been charged with the production of this disease. Many cases occur, however, which cannot be traced to this class of causes. The disease is comparatively rare among women. France gives a much larger proportion of cases than any other nation. Saloman ascribes to the female cases the depressed form of mental symptoms. In general paresis no order of symptoms is a typical one. The mental or physical symptoms may appear first, or they may be simultaneous. Parchappe reports that in eighty-six cases fifty-one were marked by the simultaneous appearance of the mental symptoms and paresis; in twenty-seven cases the paresis was subsequent, and in eight the precedence could not be determined. Leidesdorf relates the history of a case in which the earliest symptoms were spinal. On this point Maudsley says: "Before asserting in a particular case that there is no evidence of paralysis, it will be well to observe the patient when emotionally excited, or after a sleepless night; then there may be exhibited a tremulousness about his speech which is not at all visible when he is perfectly calm and collected." Forbes Winslow (Obscure Diseases of the Brain and Disorders of the Mind) says, speaking of the peculiar mental symptoms of the disease: "I have known the tendency to distort facts and look extravagantly at the bright side of everything through an intensely magnified and highly-colored, because morbid, medium to exist for five or even ten years before the mind presented any decided and recognized symptoms of alienation." M. Parchappe, in an address before the Medico-Psychological Society at Paris, 1858, says: "Mental disorder is constant from the first, at least under the form of impairment of the memory and judgment, and very frequently under that of maniacal or melancholic excitement."

The testimony of these quotations shows how difficult is not only the decision of the precedence of the symptoms, but also the diagnosis of the disease. The mental symptoms are ushered in by a stage of melancholia; but even this bears on the exaggeration of the *ego* — the patient is boastful of bodily ailments or persecutions. Gradually the ideas become more of the *ambitieux* type; an exaggeration of all that is favorable in the man or his life, and a new and falsely-colored representation of what is unworthy. With continued loss of memory we find a diminution in the coherence of ideas, until in the later stages there is observed merely the utterance of disconnected egotisms and optimisms. Finally, these psychical symptoms are not, according to some authorities, characteristic of the disease. M. Trelat (*Annales Medico-Psychologiques*, 1855) writes: "A great number of paralytics have no ideas of riches or greatness. There are some who, instead of being powerful, happy, and full of confidence, are timid, distrustful, and agitated by hypermaniacal ideas." M. Laseque (thesis on his examination for a fellowship, quoted by M. Arch Foville, Jr., *Quarterly Journal of Psychological Medicine*, New York, April, 1872,)

says, "the characteristic delirium may be wanting in spite of the intellectual disturbance." And again, M. Linas (quoted by the same authority) declares "he could not raise his voice too energetically against the opinion which assigns to general paralysis a definite delirium."

Among the first physical symptoms calling for medical treatment may be apoplectiform or epileptiform attacks and signs of cerebral congestion. The difficulties of articulation are usually among the earlier symptoms. Articulation becomes thick and labored, and loses distinctness and precision. Especial difficulty is experienced in the pronunciation of the labials. In the last stages "it consists only in the muttering of thick, indistinctly-articulated noises." Saloman (translated by Moore and quoted by Delaye) accounts for this condition by the "transient convulsive movements (involuntary spasms) of the muscles of the face, especially of the upper lip," and "fibrillar convulsions in the muscles of the tongue." The pathology of this condition is the implication of the anterior lobes in the degeneration, whether such changes be considered, with Parchappe, a cerebral softening (Medico-Psychological Society of Paris, 1858,) or Delaye, a molecular change of the cerebral tissue, especially of the gray substance (*De la paralysie generale incomplete, These de Paris*, 1822,) or Bayle, a meningitis (*Recherches sur les Maladies Mentales*, Paris, 1822,) or Behomme (quoted by Parchappe,) a meningo-cerebritis, or Calmeil, a chronic diffused periencephalo-meningitis (*De la paralysie consideree chez les Alienes*, Paris, 1822.)

It is of importance to remember that the condition of the limbs is not one of palsy, where the muscles refuse to respond to the mandate of the nervous centres; but, as the late Dr. Skae, of the Morningside Asylum of Scotland, states it (*Edinburgh Medical Journal*, April, 1860,) "the volition is irregularly conveyed and distributed. The person cannot control and direct his movements perfectly and consentaneously, just as a drunken man sees double because he cannot converge upon a given object, or walks unsteadily because he cannot direct and regulate the harmonious movements of his limbs." Dr. Reynolds, in his treatise on wasting palsy, states the means of diagnosis between general paresis and wasting palsy to be that in general paresis the muscles respond to the electrical stimulus, and in wasting palsy they do not. The functions of sight and hearing are not in ordinary cases involved except in the last stages. Smell and taste are much oftener affected, so that, according to Saloman, "the patient submits to their operation the most loathsome things." Hallucinations (endogenous sensations) are not unfrequently met with in sight and hearing. Hyperæsthesia and anæsthesia of the skin and mucous membranes are occasional but not essential symptoms of the disease. M. Parchappe noticed a slight increase of the frequency of the pulse in general paresis, although the disease, except accidentally in the state of congestion, is not accompanied by a true febrile movement. In the last stage, a condition called by M. Parchappe "cerebral marasmus," in which occur pyæmia, pneumonia, colliquative diarrhœa, gangrenous destruction of tissue from effects of pressure, terminates the sad history. All these symptoms are subject to ameliorations, which to the inexperienced lend false hopes of recovery.

The prognosis is always *unfavorable.* Parchappe says: "I have not obtained a single positive and certain cure." Dr. G. Mackenzie Bacon (*Journal of Mental Science,* London, July, 1871,) declares: "Whatever the hopes of the therapeutics of the future, I can see no reason to wander through the pharmacopœia in search of a drug to cure a disease which depends essentially on structural changes, when we know that these, at least in the brain, are not remedied by medicines."

In the treatment of general paresis, tonics and stimulants are always of use, with sedatives or narcotics when necessary.

Parchappe, as a fair exponent of the French alienists, says of the pathology of the disease: "In the majority of cases the softening (which he considers the characteristic lesion) is found at the surface on the free margin of the convolutions. At a later period it often attacks the gray matter of the corpora striata, optic thalami, medulla spinalis, and cerebellum. Mental disorder, under the form of mania or melancholia, coincides with the superficial alteration of the cortical substance; the paresis and dementia are connected with the deeper alterations, the difficulty of speech with the lesion of the anterior lobes. A greater degree of softening is often found on one side, then the paresis is more determined on the other." In America the only investigations of importance have been begun at the New York State Asylum, by its special pathologist, Dr. E. R. Hun, of Albany, N. Y. In his last report he speaks of the autopsies in cases of general paresis: "Of the eight cases of general paresis six were men and two women, the oldest sixty-four and the youngest thirty-three years of age. In five cases there were eschars or ill-conditioned ulcers observed upon the surface of the body; the skull-cap was unusually thick in four cases; the membranes were adherent to each other in four cases; the arachnoid opaque in four and congested in one; a considerable amount of sub-arachnoid serous effusion was noticed in seven of the eight cases, and in two cases clots of blood were found upon the surface of the brain. In six of the eight cases there was more or less atrophy of the cerebral convolutions, and in one case embolism of the middle cerebral artery was accompanied by softening of the hemispheres. In this case two small cysts, filled with clear serous fluid, were found encroaching upon the brain substance. The weight of the encephalic mass (including cerebrum, cerebellum, pons, and medulla) was taken in three cases; the heaviest weighed 48¼ ounces and the lightest 35¼ ounces, the average weight being 43⅓ ounces. In five of the cases there was existing tubercular disease, or evidences that such disease had formerly existed, as shown by cicatrices and cretaceous deposits in the lungs. In one case ossification of the mitral valve was found, and in three was marked deflection of the transverse colon toward the symphysis pubis."—*Am. Practitioner.*

